# Keeping All the PIECES: Phylogenetically Informed *Ex Situ* Conservation of Endangered Species

**DOI:** 10.1371/journal.pone.0156973

**Published:** 2016-06-03

**Authors:** Daniel J. Larkin, Sarah K. Jacobi, Andrew L. Hipp, Andrea T. Kramer

**Affiliations:** 1 Department of Fisheries, Wildlife, and Conservation Biology, University of Minnesota, St. Paul, Minnesota, United States of America; 2 Plant Science and Conservation, Chicago Botanic Garden, Glencoe, Illinois, United States of America; 3 Herbarium, The Morton Arboretum, Lisle, Illinois, United States of America; 4 Department of Botany, The Field Museum, Chicago, Illinois, United States of America; University of Florida, UNITED STATES

## Abstract

*Ex situ* conservation in germplasm and living collections is a major focus of global plant conservation strategies. Prioritizing species for *ex situ* collection is a necessary component of this effort for which sound strategies are needed. Phylogenetic considerations can play an important role in prioritization. Collections that are more phylogenetically diverse are likely to encompass more ecological and trait variation, and thus provide stronger conservation insurance and richer resources for future restoration efforts. However, phylogenetic criteria need to be weighed against other, potentially competing objectives. We used *ex situ* collection and threat rank data for North American angiosperms to investigate gaps in *ex situ* coverage and phylogenetic diversity of collections and to develop a flexible framework for prioritizing species across multiple objectives. We found that *ex situ* coverage of 18,766 North American angiosperm taxa was low with respect to the most vulnerable taxa: just 43% of vulnerable to critically imperiled taxa were in *ex situ* collections, far short of a year-2020 goal of 75%. In addition, species held in *ex situ* collections were phylogenetically clustered (*P* < 0.001), i.e., collections comprised less phylogenetic diversity than would be expected had species been drawn at random. These patterns support incorporating phylogenetic considerations into *ex situ* prioritization in a manner balanced with other criteria, such as vulnerability. To meet this need, we present the ‘PIECES’ index (Phylogenetically Informed *Ex situ* Conservation of Endangered Species). PIECES integrates phylogenetic considerations into a flexible framework for prioritizing species across competing objectives using multi-criteria decision analysis. Applying PIECES to prioritizing *ex situ* conservation of North American angiosperms, we show strong return on investment across multiple objectives, some of which are negatively correlated with each other. A spreadsheet-based decision support tool for North American angiosperms is provided; this tool can be customized to align with different conservation objectives.

## Introduction

*Ex situ* conservation of plant species in germplasm and living collections is increasingly used to ensure against extinction and provide materials for future restoration efforts [1–3]. Securing more of the world’s flora in *ex situ* collections is a key component of global plant conservation efforts. In 2009 the Global Strategy for Plant Conservation (GSPC) set a target that 75% of the world’s threatened flora be held in *ex situ* collections by 2020 and that at least 20% of these collections be available for restoration programs [4]. Yet in 2014, 46% of threatened species were still missing from *ex situ* collections [5]. Reaching ambitious targets will require significant investment and coordination, as e*x situ* conservation can be costly and difficult to implement.

Historically, criteria for prioritizing species for *ex situ* conservation have included factors such as endangerment status, geography, and cultural or economic value [3], as well as ability to acquire and maintain genetically diverse material in a collection [6]. In the race to achieve global *ex situ* targets, species may be prioritized simply by their absence in current collections. Recently there have been calls to consider phylogeny (evolutionary relationships among species) in *ex situ* prioritization efforts [2, 7]. Closely related species are often more similar to each other in their traits than expected by chance (i.e., phylogenetically clustered) [8]. For example, species that are more closely related may be more similar in their responses to environmental change [9–12], functional traits [13, 14], and cultural or economic value [15, 16]—though there are certainly counter examples where phylogeny fails to represent key differences among species [17, 18]. In general, phylogenetic conservatism is likely to become more pronounced as phylogenetic scale increases and a greater number of traits are considered [8]. Thus, *ex situ* collections that are individually or collectively broad in their representation of the ‘Tree of Life’ are likely to capture a wider array of trait variation [19], increasing their value for future uses like ecological restoration [19–24].

Identifying and implementing the most appropriate prioritization criteria to incorporate phylogenetic considerations into building *ex situ* collections can be challenging, and several recent studies have investigated the justification for and potential impacts of incorporating phylogenetic criteria. Isaac et al. [25] proposed that species that are more endangered and represent more unique evolutionary history should be given higher priority for conservation. They introduced evolutionary distinctiveness (ED), a measure of species’ relative contributions to phylogenetic diversity, and integrated this information with extinction risk to produce ‘EDGE scores’ identifying mammals [25] and amphibians [26] that are both “Evolutionarily Distinct and Globally Endangered.” Griffiths et al. [7] used a similar approach to prioritize legume species for seed banking, first identifying species absent from the Millennium Seed Bank and then prioritizing those gap species based on their evolutionary distinctiveness.

Given that information on the evolutionary history of species can help *ex situ* collection managers build more robust and diverse collections, phylogenetic information is likely to play an increasing role in decision-making for building and maintaining *ex situ* collections [2, 7]. However, phylogeny needs to be weighed against other considerations, and its overemphasis could be counterproductive depending on the goals of the *ex situ* collection. For example, vulnerability is often phylogenetically clustered [27], as has been shown for plants in New England (USA) [10] and South Africa’s Cape region [28]. Where this is the case, an *ex situ* program seeking to maximize phylogenetic diversity is likely to under-sample the species most in need of protection. This illustrates that phylogenetically based recommendations might differ depending, for example, on whether they are used to target what Guerrant et al. [2] described as “insurance policies against extinction” or “working capital for restoration.” Phylogenetic information should be balanced against other criteria and its use customized to serve the particular goals of a given *ex situ* program.

**Fig 1:**
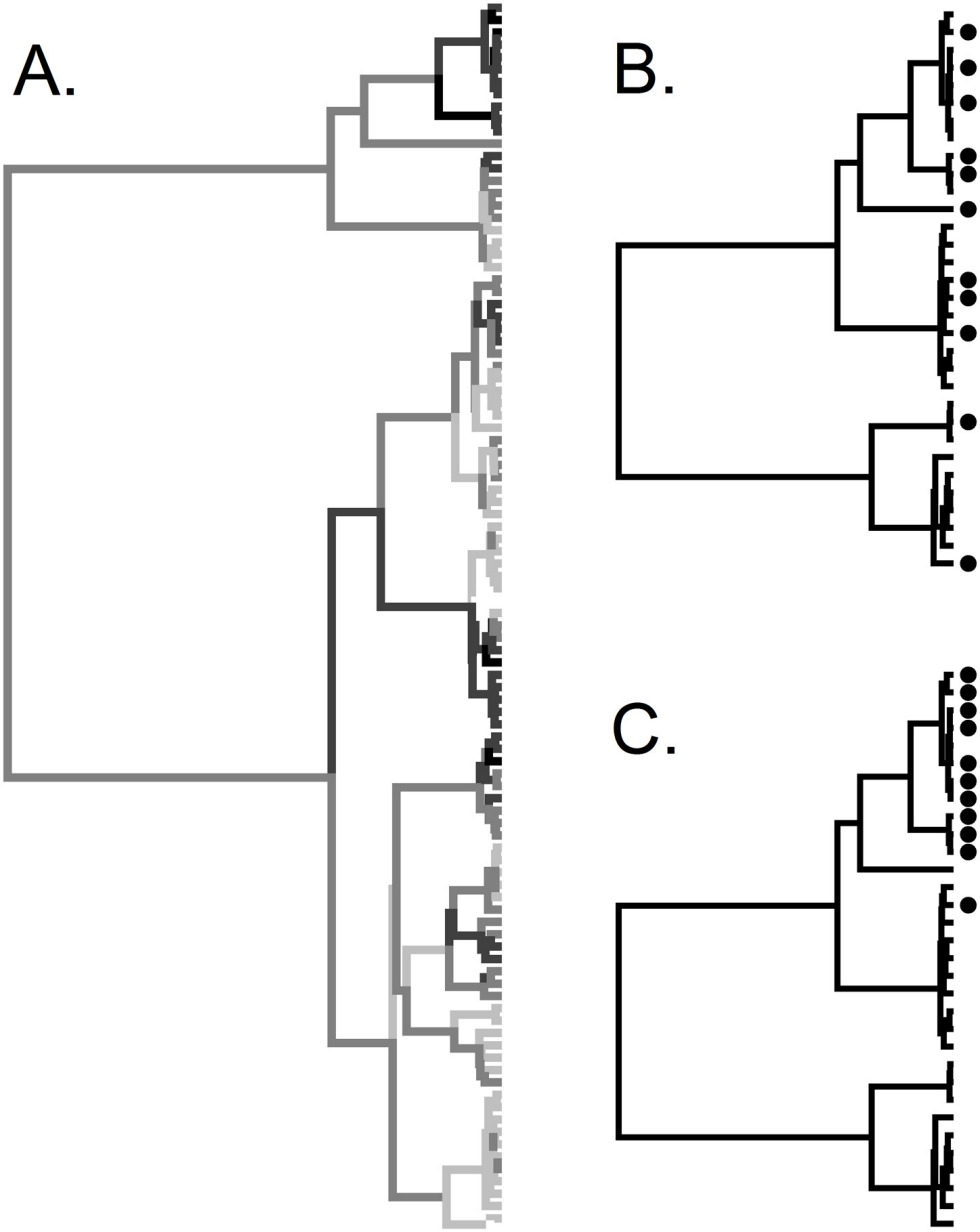
A hypothetical phylogeny of 100 plant species. (A) Darkness of branches is proportional to species’ vulnerability and shows strong phylogenetic signal (clustering), with species that are more (or less) at risk being more closely related than expected by chance. (B) A subtree showing the 32 most vulnerable species from the full tree, of which 11 are found in *ex situ* collections (black circles). If these 11 collected species were compared to the full tree, they would be identified as clustered. However, they are randomly distributed in relation to the subset of vulnerable species. (C) Same subtree as (B) but with a different distribution of *ex situ* species. Here the collected species are not only clustered with respect to the full tree but are also a phylogenetically biased subset of vulnerable species.

We used data on North American angiosperms to investigate phylogenetic aspects of building and managing *ex situ* collections and to develop a flexible framework for phylogenetically informed species prioritization in pursuit of multiple conservation objectives. We first performed gap analyses to assess current coverage of North American *ex situ* collections and coverage with respect to vulnerability and phylogeny. If vulnerable species are phylogenetically clustered (Fig 1A), then perhaps *ex situ* collections should be correspondingly clustered. However, within the subset of vulnerable species, there would ideally not be phylogenetic bias in which of those species are conserved (Fig 1B). If there is phylogenetic bias (Fig 1C), collections will have deficits in terms of evolutionary diversity and, likely, trait diversity. Thus, our phylogenetic analyses comprised sequential tests to determine whether vulnerable species were phylogenetically clustered, whether *ex situ* collections as a whole were clustered, and whether there was phylogenetic bias with respect to which vulnerable species were held in *ex situ* collections.

**Fig 2:**
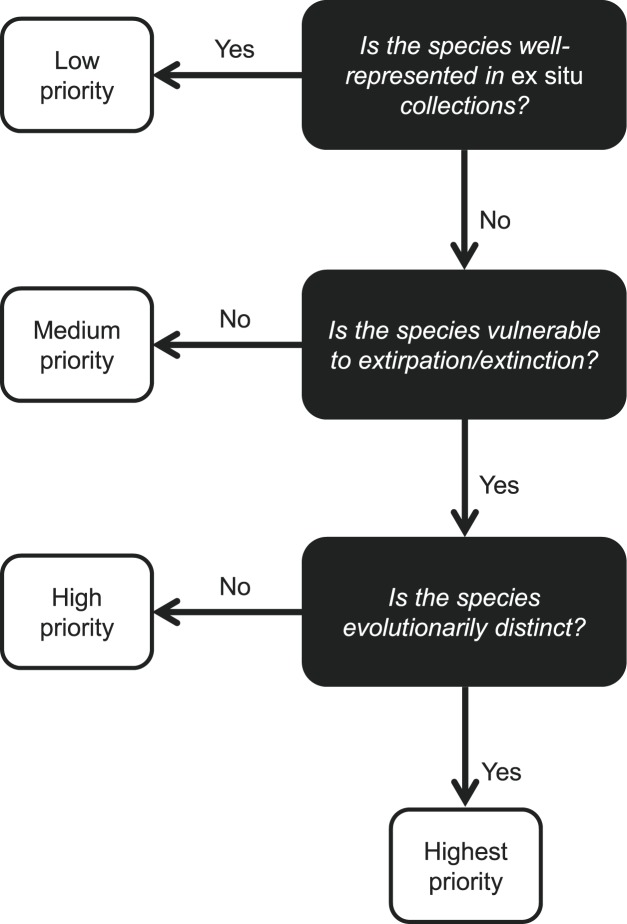
Decision tree for prioritizing species for *ex situ* collection based on their conservation status, vulnerability, and evolutionary distinctiveness.

We found that *ex situ* coverage was low with respect to the most vulnerable species, vulnerability was phylogenetically clustered, and extant *ex situ* collections were clustered, underrepresenting both the full phylogenetic tree of North American angiosperms and subtrees of vulnerable species. These patterns support incorporating phylogenetic considerations into *ex situ* prioritization, but the relative weight to place on phylogeny relative to other prioritization criteria remains a question. To navigate this challenge, we propose an approach that favors species that are least protected and most vulnerable, and then prioritizes species to increase collections’ phylogenetic diversity (Fig 2). For this, we developed the ‘PIECES’ index (Phylogenetically Informed *E**x situ* Conservation of Endangered Species), which uses multi-criteria decision analysis to integrate phylogenetic considerations with other objectives in order to prioritize collection of new taxa. We then applied PIECES to selecting North American angiosperms for addition to *ex situ* collections and compared return on investment (ROI) for different conservation objectives using PIECES and four alternative prioritization approaches. We also evaluated overlap in taxonomic and phylogenetic composition of the taxa selected using these five approaches to determine how similar or different their solution sets were. To make the outcomes of this work adaptable to the objectives of different conservation programs, we include a spreadsheet-based decision support tool that can be modified to align with different priorities for *ex situ* conservation of North American angiosperms.

## Materials and Methods

### North American *ex situ* collections

We assembled our species list using NatureServe Explorer [29], a biodiversity and conservation database, which follows Kartesz’s [30] taxonomy. Target taxa (species, or subspecies where applicable) were restricted to North American angiosperms in this database (*N* = 21,555). Conservation status (threat rank) was standardized across NatureServe’s Global Conservation Status Ranks, resulting in all taxa being placed into one of nine categories: R1-critically imperiled, R2-imperiled, R3-vulnerable, R4-apparently secure, R5-secure, non-native, extinct, unrankable, or subsumed through taxonomic revision. NatureServe ranks are the most complete threat assessment available for North American vascular plants; the protocol used is similar to that employed by IUCN, but coverage is much more complete [29, 31].

The taxa list was then reduced to those ranked R1 through R5 (*N* = 18,766) and cross-referenced with the PlantSearch database of Botanic Gardens Conservation International [32], which comprised approximately 1.3 million records for living, seed, and tissue collections from over 1,000 institutions. For each ranked taxon, the BGCI database was used to determine *ex situ* status in terms of numbers of living collections and germplasm collections (both seed and tissue culture, hereafter “seed bank”). We also evaluated whether *ex situ* coverage differed by threat rank using a chi-squared test of the number of taxa of each rank recorded in seed banks, living collections, both, or neither. This test was performed in R version 3.1.1 [33].

### Phylogenetic analysis

We modified a dated molecular phylogeny of 32,223 taxa of land plants published by Zanne et al. [34, 35] to construct a tree for our focal taxa. The Zanne et al. phylogeny was constructed based on GenBank sequence data for seven gene regions (18S rDNA, 26S rDNA, ITS, matK, rbcL, atpB, and trnL-F) using maximum likelihood for tree estimation [34, 36]. Nomenclature for our dataset and the Zanne et al. tree was reconciled using the Taxonomic Name Resolution Service [37, 38]. Of our focal taxa, 4,058 (22.5%) were present in the Zanne et al. phylogeny. The remainder were primarily added as genus-level polytomies (i.e., not fully resolved to dichotomous splits, with three or more sister taxa descending from a single node) using the ape and phytools packages in R [39, 40], with branch lengths for added taxa set at the crown depths for their respective genera. Some taxa lacked congeners in the tree (*N* = 436); these were placed as polytomies at the crowns for their families. Finally, all non-target taxa were dropped, resulting in a phylogeny containing only the 18,766 focal taxa. See Supporting Information (S1 Phylogeny) for a Newick-formatted file of this tree. Phylogenetic resolution—the ratio of the number of nodes to that which would be found in a fully resolved phylogeny [41]—was low, 26%, due to polytomies. However, polytomies only occurred at terminal branches, deeper nodes were fully resolved, and we evaluated the sensitivity of our analyses to effects of polytomies (see below).

We tested for phylogenetic signal—closer relatives being more similar in their trait values—using Fritz and Purvis’ [41] *D* statistic for binary traits. If a binary trait (e.g., taxa being present or absent in seed banks) is phylogenetically random, then *D* = 1; while for a trait as conserved (clustered) as expected under a Brownian motion model of evolution, *D* = 0 [41]. For each trait, we estimated *D* and tested whether it was non-random (*D* < 1) or as clustered as expected under a Brownian model (*D* = 0) using the ‘phylo.d’ function in the R package caper [42], with 1,000 permutations to assess significance. We used the full tree to test whether there was phylogenetic signal in taxa’s threat ranks, and with respect to which taxa were found in seed or living collections. Having found that there was phylogenetic signal with respect to threat ranks, subtrees comprising only taxa of a given threat rank were used to test for phylogenetic bias in which of those taxa were in collections. For example, a tree containing only the 1,490 R1 taxa was used to determine whether the 470 R1 taxa found in seed banks were phylogenetically clustered. Were the full tree to be used for this test, detection of biases in collections by threat rank would be confounded by phylogenetic patterns in vulnerability (see Fig 1).

Polytomies can exaggerate appearance of phylogenetic conservatism (false positives) [43], weaken detection of conservatism (false negatives) [44], or have little effect on phylogenetic signal or its uncertainty [45]. The direction and magnitude of these effects vary with polytomies’ size and phylogenetic depth [43, 45]. We tested whether terminal polytomies in our tree were likely to bias our results. We began with a fully resolved phylogeny comprising a subset of our focal taxa (the 4,058 taxa found in the Zanne et al. tree) and then generated trees of decreasing phylogenetic resolution by collapsing terminal branches of increasing length into polytomies using the ‘di2multi’ function in ape [39]. This yielded 20 trees spanning from 100% to <1% phylogenetic resolution. For each tree, we tested for phylogenetic signal in threat ranks and seed and living collections as described above. We found that ability to detect phylogenetic signal gradually decreased with decreasing phylogenetic resolution (S1 Fig). However, within the range of 25% to 100% resolution, interpretation was the same for all traits (phylogenetically non-random but less clustered than under a Brownian model) except for status as an R2 species, which changed from being phylogenetically non-random to random below 70% resolution. Thus we consider tests for phylogenetic signal using the 26%-resolved tree to be reasonable but likely conservative.

### Developing the PIECES score

*Ex situ* conservation is intended to yield returns for multiple objectives, e.g., increased protection of endangered species and of evolutionarily underrepresented species. Optimizing for a single objective is straightforward, but solving for multiple goals is complex [46], leading to decisions for *ex situ* conservation involving tradeoffs among competing objectives. For example, the sets of candidate taxa for *ex situ* conservation that are least collected, most endangered, or would do the most to increase collections’ phylogenetic diversity are likely to differ. To address these tradeoffs in a systematic manner, we considered the selection of taxa as a multi-criteria decision analysis (MCDA) problem, a decision approach used to deal with tradeoffs among multiple objectives [47].

We employed MCDA in developing the PIECES score, a flexible means of prioritizing taxa for new additions to *ex situ* collections. PIECES builds upon existing prioritization schemes while incorporating additional considerations. Specifically, our formulation of PIECES includes parameters for insufficient *ex situ* coverage, encompassing both seed banks [as in 7] and living collections, which have not been incorporated into previous approaches to our knowledge; threat rank, a component of the EDGE score of Isaac et al. [25]; and evolutionary distinctiveness [included in 7, 25]. We created an objectives hierarchy to organize these criteria, and relative-preference weighting of objectives was performed using MCDA (see below). This is intended as an adaptable approach to prioritization: parameters and their weights can be modified to meet the objectives and constraints of different conservation programs.

PIECES scores were calculated as follows:

$$PIECES\;=\;100\;\times \left(w_{1}SB\;+\;w_{2}LC+\;w_{3}GE+\;w_{4}ED\right)$$

Where values for component variables were normalized from 0 to 1 and the sum of all weights (*w*_i_) equals 1. This formula results in possible scores for taxa ranging from 0 (lowest priority for *ex situ* conservation) to 100 (highest priority).

SB (Seed Bank) and LC (Living Collections) represent deficits in *ex situ* coverage based on the number of seed bank and living collections for each taxon recorded in the BGCI database [32]. These variables were first log-transformed (ln[*x* + 1]) and then scaled in reverse from 1 (no seed or living collections) to 0 (maximum number of collections recorded across all species). SB and LC were not treated as binary (present or not in collections) because multiple collections of a taxon increase its security and provide better coverage across genotypes/ecotypes [2]. Log transformation was used to account for non-linearity in the relative value of additional collections, i.e., each new collection for a taxon is relatively more important when there are few collections and returns diminish as collections increase. GE [Globally Endangered, sensu 25] indicates threat ranks, scored as R1 = 1, R2 = 0.75, R3 = 0.5, R4 = 0.25, and R5 = 0. ED (Evolutionary Distinctiveness) measures a taxon’s contribution to overall phylogenetic diversity based on the unique evolutionary history (branch length) that it represents. ED was calculated using the ‘ed.calc’ function in the R package caper [42], with the ‘Isaac correction’ used to account for polytomies, which otherwise inflate ED estimates [25]. ED was then scaled from 0 to 1 (least to most evolutionarily distinct).

The relative weights assigned to each component of PIECES dictate the influence of that component on prioritization. Determining weights that yield results balanced across objectives was a challenging problem for which we employed MCDA. MCDA distills the problem into distinct components: problem objectives, alternatives used to achieve objectives, evaluation of each alternative’s performance on each objective, and the decision maker’s relative preference for each objective. As described above, our objectives with PIECES were to increase *ex situ* coverage while favoring taxa that are threatened and evolutionarily distinct. We gave greater weight to taxa missing from seed banks than those missing from living collections, as seed bank collections generally provide more conservation and restoration value than living collections [3], though this is not true for certain groups of plants, such as oaks [48]. Alternatives for achieving our objectives were represented by combinations of subsets of taxa that could be chosen for *ex situ* conservation.

We assigned preference weights for each objective using the Analytical Hierarchy Process (AHP) [49]. AHP begins with creation of an objectives hierarchy that decomposes the problem into more manageable sub-problems. The decision maker is then asked to make pairwise comparisons regarding the relative importance of each sub-objective with respect to its grouping. The decision maker chooses values on a scale of 1–9, where 1 indicates that both objectives are equally important and 9 indicates that one objective is far more important than the other. After all pairwise comparisons have been considered, AHP computes the relative weights for each objective and sub-objective in the hierarchy. This is accomplished by normalizing the principal right eigenvector of the matrix containing all pairwise comparisons. The resulting eigenvalue is a vector of relative weights from 0–1, the sum of which equals 1. In addition to preference weights, the decision maker is also asked to select the number of taxa to add to *ex situ* collections (*n*). The identities of the taxa to include are found by computing the PIECES score for each taxon based on the assigned preference weights, and selecting the top *n*-ranked taxa.

We developed a tool in Microsoft Excel (Redmond, Washington, USA) that performs AHP as described above (S1 Appendix). The decision maker is guided through the process of evaluating each pairwise combination of objectives. The resulting weights and objective values are displayed, along with the taxa selected. By iteratively refining preference weights, decision makers can ensure that the objective values achieved align with their conservation objectives. In addition, the pool of species to choose from can be modified by selecting which taxa to include or exclude as candidate species. The species table contains pertinent information that can be filtered or sorted by to aid this process, e.g., family, threat rank, etc.

### Conservation return on investment for alternative prioritization schemes

We characterized the conservation return on investment (ROI) for PIECES and other solutions to the hypothetical problem of prioritizing 1,000 North American angiosperm taxa for *ex situ* collection—an ambitious but plausible target. ROI was quantified based on the objectives included in the PIECES index. Specifically, for the 1,000 taxa selected, we counted the number that comprised novel additions to seed banks or living collections and calculated their mean threat ranks and evolutionary distinctiveness.

We used a multi-step approach to quantify and contextualize ROI. First, we characterized the *potential* distribution of ROI for each objective using a “weight space analysis” [50]. By choosing different preference weights, different possible outcomes for the various objectives can be achieved. To investigate the range of possibilities across objectives, i.e., the weight space, we created 100,000 randomly generated vectors of preference weights for the components of PIECES such that ∑*w*_i_ = 1. For each of these vector weights, we selected the 1,000 highest-ranked taxa (per that weighting of objectives) and calculated ROI for each objective. To identify potential tradeoffs among objectives, we plotted all pairwise correlations between objectives using results from the weight space analysis. We also identified the Pareto frontier for each pairwise comparison of objectives—the values of each objective such that one objective cannot be improved without deteriorating the other objective [51]—using the ‘Pareto front’ function in Matlab [52]. These visualizations show the outcomes available across possible weight combinations, highlight tradeoffs among objectives, and can be used to refine perceptions of acceptable outcomes [50].

Second, we evaluated how the ROI for PIECES and other prioritization schemes fared in relation to the full weight space and to one another. In addition to PIECES, we included four schemes that prioritized taxa based solely on their: 1) threat rank (Endangered); 2) absence from *ex situ* collections (Ex Situ); 3) absence from seed banks and ED, equivalent to the approach of Griffiths et al. [7] (ED + Seed Bank); and 4) threat rank and ED, as in Isaac et al. [25] (EDGE). For the Endangered and Ex Situ approaches, there were multiple equivalent solutions: any 1,000 of the 8,228 taxa not held in *ex situ* collections could be selected for Ex Situ or any of the 1,490 taxa ranked R1 could be selected for Endangered. Thus, for these models we randomly subsampled 1,000 sets of 1,000 suitable candidate taxa and calculated mean ROI across subsamples.

To assess the influence of alternative prioritization decisions on the composition of *ex situ* collections, we assessed how similar in taxonomic and phylogenetic composition the 1,000 taxa selected by different schemes were. We measured overlap in taxonomic composition as the proportion of shared taxa using the binary method of the ‘dist’ function in R. Phylogenetic similarity was measured as the proportion of branch length shared between subsets of taxa, calculated using the ‘phylosor’ function in the R package picante. For approaches with multiple equivalent solutions (Endangered and Ex Situ), similarities to other prioritizations were calculated as means across 100 randomly drawn solutions.

## Results

See Supporting Information (S1 Table) for a complete list of focal taxa, including their threat ranks, counts of extant seed and living collections, evolutionary distinctiveness, and scores and rankings for different prioritization approaches.

**Fig 3:**
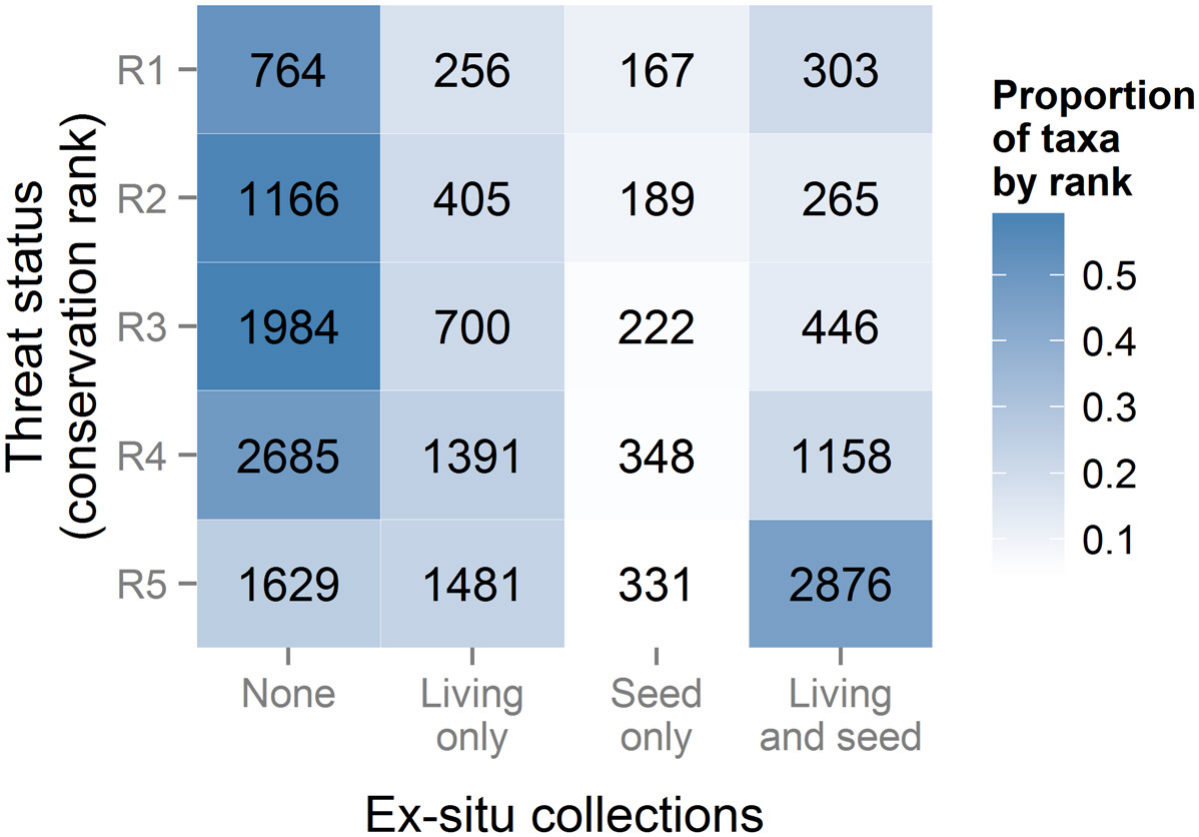
Distribution of *ex situ* collections for North American angiosperm taxa by threat rank. Numbers in cells are numbers of taxa of each collection status, which differed by threat rank (*Χ*^2^ = 2203, *P* << 0.0001). Shading indicates the proportion of taxa within a threat rank with a given *ex situ* collection status.

*Ex situ* coverage differed strongly by threat rank (*Χ*^2^ = 2203, *P* << 0.0001) (Fig 3). In general, common (R5) taxa were most likely to be in an *ex situ* collection (74%), while apparently secure and critically imperiled taxa were next most likely to be in a collection (52% and 49%, respectively), leaving threatened and vulnerable taxa least likely to be held in seed banks or living collections (41% and 42%). There was non-random phylogenetic signal in taxa’s threat ranks and *ex situ* coverage (Table 1). In all cases, these attributes were more phylogenetically clustered than expected under a random model (*P* < 0.001), but less clustered than predicted by a Brownian motion model (*P* < 0.001).

**Table 1:** Phylogenetic signal (*D* statistic) of focal taxa’s *ex situ* statuses and threat ranks.

Phylogeny	*N*_phylogeny_^a^	Category	*N*_category_^b^	*D*	*P*(*D* < 1)	*P*(*D* > 0)
Full tree	18,766	*Ex situ*–seed	6,305	0.84	< 0.001	< 0.001
Full tree	18,766	*Ex situ*–living	9,281	0.72	< 0.001	< 0.001
Full tree	18,766	Rank–R1	1,490	0.80	< 0.001	< 0.001
Full tree	18,766	Rank–R2	2,025	0.92	< 0.001	< 0.001
Full tree	18,766	Rank–R3	3,352	0.93	< 0.001	< 0.001
Full tree	18,766	Rank–R4	5,582	0.93	< 0.001	< 0.001
Full tree	18,766	Rank–R5	6,317	0.75	< 0.001	< 0.001
R1 only	1,490	*Ex situ*–seed	470	0.87	< 0.001	< 0.001
R2 only	2,025	*Ex situ*–seed	454	0.93	< 0.001	< 0.001
R3 only	3,352	*Ex situ*–seed	668	0.89	< 0.001	< 0.001
R4 only	5,582	*Ex situ*–seed	1,506	0.88	< 0.001	< 0.001
R5 only	6,317	*Ex situ*–seed	3,207	0.89	< 0.001	< 0.001
R1 only	1,490	*Ex situ*–living	559	0.63	< 0.001	< 0.001
R2 only	2,025	*Ex situ*–living	670	0.68	< 0.001	< 0.001
R3 only	3,352	*Ex situ*–living	1,146	0.68	< 0.001	< 0.001
R4 only	5,582	*Ex situ*–living	2,549	0.76	< 0.001	< 0.001
R5 only	6,317	*Ex situ*–living	4,357	0.84	< 0.001	< 0.001

^a^ Number of taxa in the tree used to test for phylogenetic signal.

^b^ Number of taxa in the relevant category, e.g., for row one: 6,305 of the 18,766 focal taxa were represented in seed collections.

The variable weights we selected for PIECES through the MCDA process were: *w*_1_ (SB) = 0.4623, *w*_2_ (LC) = 0.0771, *w*_3_ (GE) = 0.1979, *w*_4_ (ED) = 0.2627.

**Fig 4:**
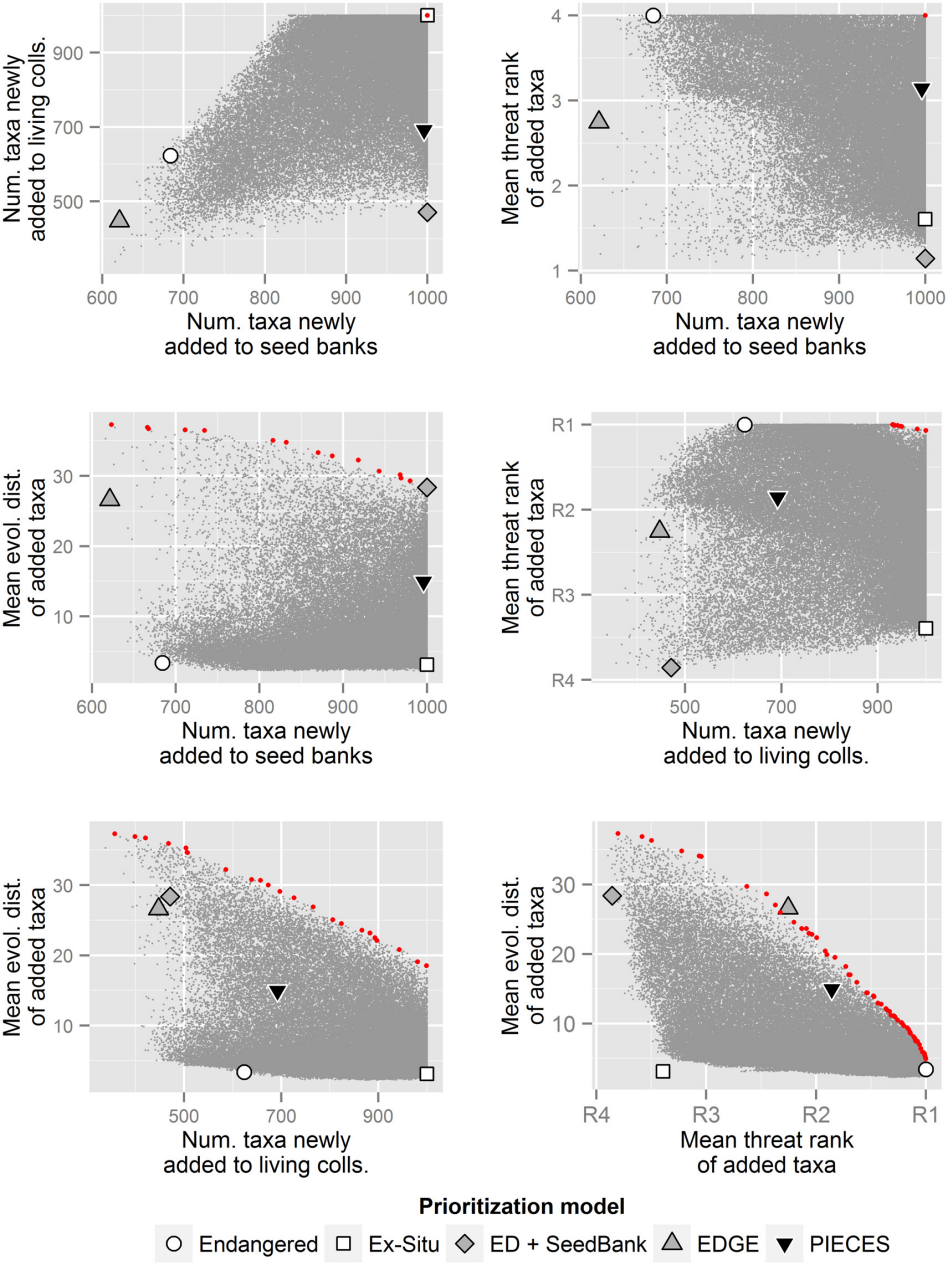
Return on investment (ROI) and tradeoffs among different objectives associated with *ex situ* conservation of 1,000 taxa selected using alternative prioritization schemes. Clouds of small gray points depict potential ROI; these were derived using weight space analysis (see Materials and Methods). Red symbols highlight the Pareto frontier: values for each objective such that one objective cannot be improved without the paired objective being deteriorated. Points indicate results for five different approaches to prioritization, with ROI as follows: Number of taxa newly added to seed banks: Endangered = 684; Ex Situ = 1,000; ED + Seedbank = 1,000; EDGE = 621; PIECES = 996. Number of taxa newly added to living collections: Endangered = 624; Ex Situ = 1,000; ED + Seedbank = 471; EDGE = 447; PIECES = 692. Mean threat rank of added taxa (where 1 = R4 [apparently secure], 4 = R1 [critically imperiled]): Endangered = 4; Ex Situ = 1.6; ED + Seedbank = 1.1; EDGE = 2.7; PIECES = 3.1. Mean evolutionary distinctiveness of added taxa: Endangered = 3.4; Ex Situ = 3.1; ED + Seedbank = 28.4; EDGE = 26.6; PIECES = 14.9.

A complex correlation structure among variables, including negative relationships, constrained how well prioritization approaches could perform across objectives (Fig 4). Alternative prioritization models differed in their conservation ROI. For each objective, one or more models performed exceptionally well relative to the overall weight space, achieving the maximum possible value. The single-factor models (Endangered and Ex Situ) only performed well for metrics directly related to threat rank and new additions to collections, respectively. The two-factor models were on the Pareto frontier for their respective objectives, i.e., ED + Seed Bank yielded optimal results for ED and new additions to seed banks and EDGE was optimal for ED and threat ranks. But these models both performed poorly with respect to other objectives. PIECES generally performed well across all ROI variables, being the only approach that was always among the top-three highest-performing models. And yet, reflecting our efforts to balance across objectives, PIECES was also the only model that never achieved the maximum possible value for any single objective.

Different prioritization schemes resulted in the selection of taxonomically and phylogenetically distinct assemblages (Table 2). PIECES and EDGE selected 40% of the same taxa. PIECES overlapped more with Ex Situ and Endangered than did the other phylogenetically informed approaches. In terms of shared phylogenetic branch length, PIECES and EDGE again had high overlap, and EDGE and PIECES were similar in their overlap with Endangered and Ex Situ.

**Table 2:** Pairwise taxonomic and phylogenetic similarity (0 = no overlap, 1 = identical) for subsets of 1,000 taxa prioritized for *ex situ* conservation using alternative prioritization schemes.

Taxonomic similarity	Endangered	Ex Situ	ED + Seed	EDGE
Ex Situ	0.115	—	—	—
ED + Seed	0.019	0.029	—	—
EDGE	0.136	0.022	0.239	—
PIECES	0.172	0.043	0.213	0.404
Phylogenetic similarity	Endangered	Ex Situ	ED + Seed	EDGE
Ex Situ	0.437	—	—	—
ED + Seed	0.297	0.277	—	—
EDGE	0.669	0.307	0.566	—
PIECES	0.651	0.343	0.562	0.720

## Discussion

For North American angiosperms, current progress toward achieving the global target for *ex situ* collections is on par with global efforts (43% of R1, R2, and R3 taxa in at least one *ex situ* collection relative to 46% globally), but this is still well short of the 2020 target of 75% [4, 5]. Likewise, 34% of all North American angiosperms are held in a seed bank or other germplasm repository, exceeding the goal of 25% set by the Millennium Seed Bank for this region. Yet *ex situ* collections of North American angiosperms contain critical gaps in coverage. Taxa that are more vulnerable are less likely to be protected in seed and living collections than common taxa, and collections are phylogenetically biased—with coverage clustered with respect to the full tree of North American angiosperms and subsets of vulnerable taxa. These patterns suggest a need for prioritization approaches that yield better returns in terms of conserving vulnerable taxa and increasing collections’ phylogenetic diversity. Our framework for addressing this need, PIECES, provides decision support that balances across multiple, sometimes competing objectives for *ex situ* conservation. This approach can be adapted to different collections priorities, aiding managers working to build and manage diverse *ex situ* collections for conservation and restoration purposes.

### Reconciling phylogenetic criteria with other objectives and constraints

Phylogenetic analysis can provide valuable insights into *ex situ* conservation, helping to identify gaps in coverage, target taxa of special concern, and provide robust resources for restoration [2, 7, 25]. However, our results show the importance of taking a careful approach to weighing phylogenetic considerations against other *ex situ* objectives. There is no “free lunch” [53]; efforts focused disproportionately on maximizing phylogenetic diversity are likely to be unsatisfactory with respect to other *ex situ* conservation goals.

We have emphasized this tradeoff in terms of vulnerability but it applies in other ways as well. Institution-based *ex situ* programs often focus on particular taxonomic groups or habitat types for scientific, geographic, or cultural reasons. Phylogenetically informed prioritization could lead to recommendations in conflict with these considerations. For example, collections for botanic gardens are often taxonomically specialized (e.g., on Cactaceae in the southwestern U.S.). In such cases, it would be a questionable strategy to target distant relatives for the sake of phylogenetic diversity alone. Instead, a phylogenetic approach in that context could involve identifying imbalances within clades of focus—as in the analysis of legume seed banks by Griffiths et al. [7]. The pool of candidate species for phylogenetically informed prioritization needs to be appropriately tailored to align with programs’ geographic locations, floristic priorities, and broader objectives.

In addition to programmatic considerations, there are biological limits to increasing collections’ phylogenetic diversity. An important caveat of our analysis is that, given our large species pool, we did not attempt to account for these limits. For example, we weighted seed bank collections more heavily than living collections, as they generally capture genetic diversity more effectively and economically, are more secure, and are more easily used in restoration programs (Guerrant et al. 2004). However, ‘exceptional’ species that do not produce seed that can be banked using traditional approaches are not candidates for seed banking. It is not yet clear just which species are exceptional, although it has been estimated that 5,000 or more endangered species will require non-seed approaches to *ex situ* conservation [54]. There are not obvious phylogenetic relationships in seed desiccation tolerance (a prerequisite for seed banking) among angiosperms [55], making it challenging to incorporate into future prioritization approaches. However, some species with desiccation-intolerant (“recalcitrant”) seeds are phylogenetically clustered, including *Quercus* (oak) species, which are disproportionately absent from seed banks despite being very prevalent in living collections. For example, *Q*. *rubra* was found in 192 living collections at the time of this assessment but only a single seedbank collection (from which it has since been removed) (S1 Table). While these species are increasingly the target of germplasm banking approaches like cryopreservation [54], living collections’ important conservation role is down-weighted in our formulation of PIECES as a result of the broad scale of our analysis.

### Insights from multi-criteria decision analysis

Use of multi-criteria decision analysis was valuable for refining our approach to prioritization. While the ROI resulting from our implementation of PIECES was consistent with our objectives hierarchy, the weights we assigned to PIECES’ component variables were not intuitive. For example, we allocated >25% of relative weight to evolutionary distinctiveness, despite its position at the bottom of our decision tree, and only 20% to threat rank, despite its higher position (Fig 2). We would have been unlikely to arrive at these weights through an *ad hoc* process.

These difficult-to-predict outcomes were a product of complex relationships among component variables (Fig 4). There were positive correlations between some objectives (e.g., novel additions to seed banks and living collections), such that allocation to one yielded an increase in the other, but negative correlations between others (e.g., threat rank and evolutionary distinctiveness). Evolutionary distinctiveness in particular was negatively correlated with several variables. Because of such tradeoffs, prioritization schemes only rarely produced results on or near the Pareto frontiers for pairs of objectives.

PIECES did not produce Pareto-optimal results in our analysis of ROI, nor did it achieve the maximum possible value for any single objective (Fig 4). However, PIECES also never performed poorly with respect to any objective. While it selected species with lower mean evolutionary distinctiveness than the other phylogenetically informed approaches, it outperformed the EDGE model in addressing deficits in *ex situ* collections and the ED + Seed Bank model in addressing threat rank (factors which we note these models were not intended to address: the EDGE model was not developed for *ex situ* collections [25] and the ED + Seed Bank approach was applied to a flora lacking systematic threat assessments [7]). The way we weighted PIECES’ component variables made it a generalist or “jack of all trades” model—pretty good at everything, but not exceptional at achieving any single objective.

It is interesting to note some of the solutions that PIECES produced to balance across objectives. For example, our highest weighted objective was novel additions to seed banks. Despite this, some of the taxa selected in our analysis of ROI (4 out of 1,000) were already found in seed banks: *Gunnera petaloidea*, *Harperocallis flava*, *Kalmiopsis fragrans*, and *Xylosma crenata*. However, these four species were each only represented in a single seed bank; *H*. *flava*, *K*. *fragrans*, and *X*. *crenata* are R1 species with high evolutionary distinctiveness (32.5–72.5); and *G*. *petaloidea* is an R2 species that was one of the 0.1% most evolutionarily distinct species in our analysis (104.3). Thus, selection of these four species was highly efficient; it required a small sacrifice in terms of a primary objective, but yielded exceptionally high returns with respect to other objectives.

Assignment of preference weights required an iterative process to balance tradeoffs across correlated responses. Because of this we designed our decision support tool (S1 Appendix) to show in real-time how changes in variable weights influence ROI. This visualization of the effects of weight choices enables an interactive approach, which helps in dealing with interactions among criteria and can improve the overall efficiency of decision making [47].

### Implications for practice

It is our hope that *ex situ* programs will consider using PIECES or other phylogenetic multi-objective approaches [see 7, 25] for prioritization support. The approach we have developed is intended to be adaptable to programs’ specific objectives. For example, updated information on taxa’s threat status or presence in *ex situ* collections can be incorporated, the species pool can be subset to include only desired taxa for a given *ex situ* program, and taxa could be scored in terms of cultural, economic, or ecological criteria and those scores incorporated into expanded versions of PIECES.

One useful enhancement would be to factor species-specific costs in to prioritization. These could be financial or effort costs associated with acquiring and maintaining *ex situ* collections of a given species [56]. For example, costs are likely to be higher for taxa that are difficult to sample, have low fecundity, or are hard to preserve. Incorporating these differences in to prioritization would enable tradeoffs to be explicitly accounted for in decision making, e.g., the opportunity costs in terms of total coverage resulting from resources being allocated to difficult taxa.

The results of any prioritization approach, including PIECES, are only as good as the data underlying it. In regions where threat status is not well known, or where it is rapidly changing, it may be impossible to incorporate this consideration into prioritization [7]. The United States is fortunate in that all native species have been run through standardized threat status assessments by NatureServe and its network of state natural heritage programs. However, as botanical capacity within states declines, and as threats to the survival of plant species grow, there is a risk that these assessments will be out of date for some taxa. In other regions of the world, the IUCN Red List is the gold standard, but plants are woefully underrepresented: as of 2009 fewer than 4% of plant species had been assessed following IUCN Red List criteria, and only two groups of plants (conifers and cycads) had been assessed in a comprehensive way [57]. Since that time, only an additional 1% of species have been assessed by IUCN [31]. Likewise, in regions where the full extent of species available in *ex situ* collections of different types is unknown or poorly understood, the approach of targeting taxa based on their current representation will not be effective. This illustrates the power of large-scale efforts to compile and synthesize *ex situ* data to inform strategic plant conservation activities, such as BGCI’s PlantSearch database [32].

Our large-scale analytical approach and dataset is perhaps best applied at the national level to help guide collaborative activities across institutions building *ex situ* collections. Ideally there will be coordination among *ex situ* programs to prioritize filling of phylogenetic and other gaps. This would allow individual programs to continue to specialize their collections based on taxonomy, geography, or other criteria, while collectively addressing deficits in coverage in an efficient manner.

## Supporting Information

S1 Appendix Spreadsheet tool (Microsoft Excel) for implementing and modifying PIECES. (XLSM)

S1 Fig Test of the effects of incompletely resolved phylogenies on detection of phylogenetic signal using the *D* statistic. (TIF)

S1 Phylogeny Phylogeny of 18,766 North American angiosperms used for phylogenetic analyses. (TRE)

S1 Table Table of all focal taxa, with values for PIECES’ component variables and ranks based on different approaches to prioritization. (CSV)
