# Supplementary figures and images for: Keeping All the PIECES: Phylogenetically Informed Ex Situ Conservation of Endangered Species
Source: PLoS One. 2016 Jun 3;11(6):e0156973. doi: 10.1371/journal.pone.0156973 (PMC4892560; doi:10.1371/journal.pone.0156973)

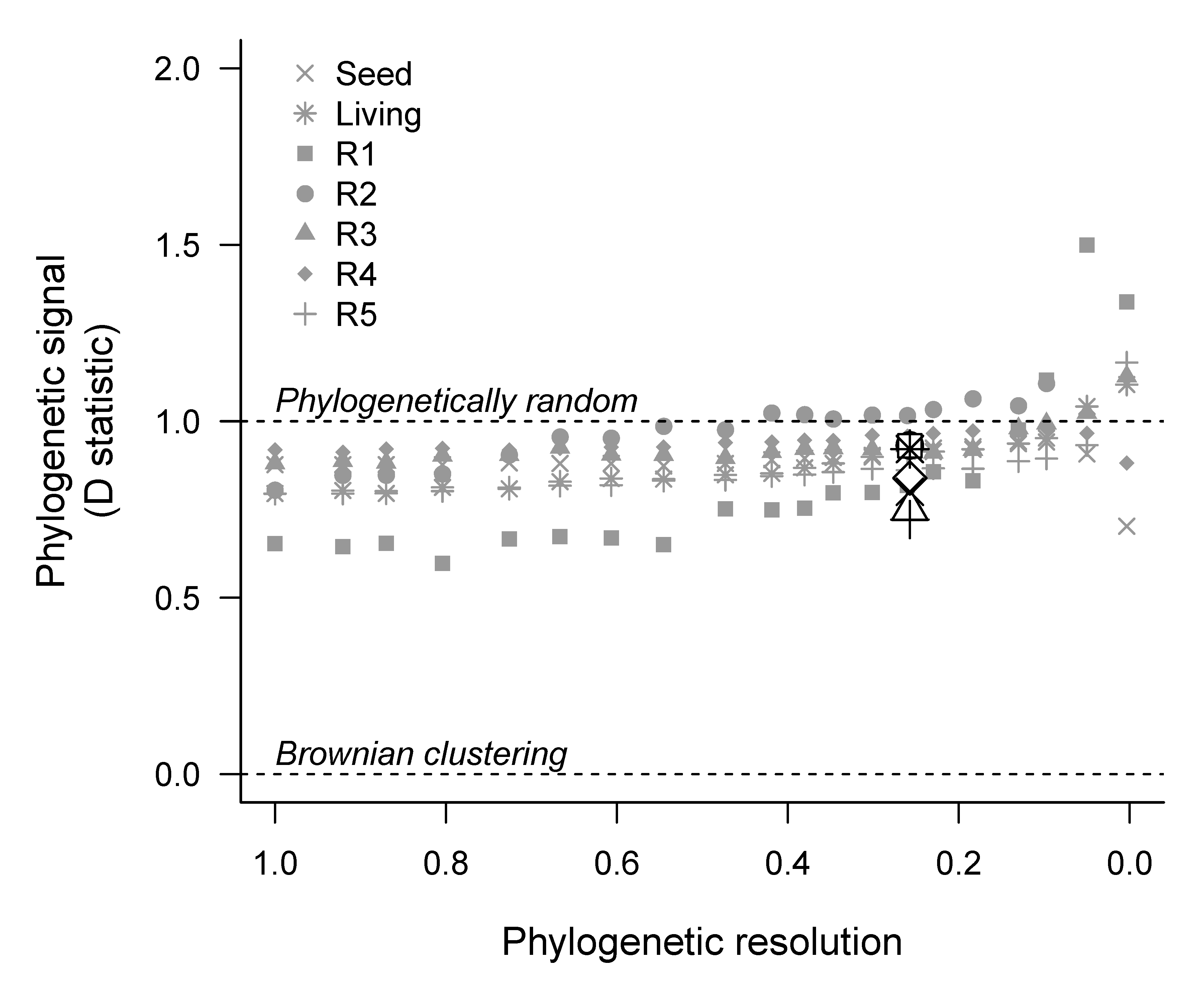

Supplement: S1 Fig — (TIF) [file pone.0156973.s002.tif]
